# Ruptured Popliteal Artery Aneurysm

**DOI:** 10.1055/s-0041-1739484

**Published:** 2021-12-28

**Authors:** Umberto G. Rossi, Francesco Petrocelli, Maurizio Cariati

**Affiliations:** 1 Interventional Radiology Unit, Department of Radiological Area, E.O. Galliera Hospital, Genova, Italy; 2 Diagnostic and Interventional Radiology Unit, Department of Diagnostic and Therapeutic Advanced Technology, Azienda Socio Sanitaria Territoriale Santi Paolo and Carlo Hospital, Milano, Italy; 3 Department of Radiology and Interventional Radiology, Istituto di Ricerca a Carattere Clinico e Scientifico San Martino Policlinic University Hospital, Genova, Italy

**Keywords:** popliteal artery, aneurysm, imaging, aging, endovascular

## Abstract

Rupture of a popliteal artery aneurysm is an uncommon event in an uncommon disease. We present the case of an 88-year-old female with a ruptured popliteal artery aneurysm that was diagnosed by multidetector computed tomography and treated by an endovascular approach.

An 88-year-old female, with arterial hypertension, was admitted to our hospital for onset of acute pain and swelling behind her left knee. There was no history of trauma. Physical examination revealed a palpable and pulsatile mass in the upper popliteal fossa. Left ankle brachial index was 0.8 (normal range: 0.9–1.2). Multidetector computed tomography of left lower limb, on axial and sagittal volume rendering technique reconstruction, revealed a voluminous popliteal artery aneurysm (4.1 cm; arrowhead) with partial aneurysm sac thrombosis and signs of contained rupture ( Fig. 1A, B ). She was felt to be a candidate for an urgent percutaneous endovascular approach.

**Fig. 1:**
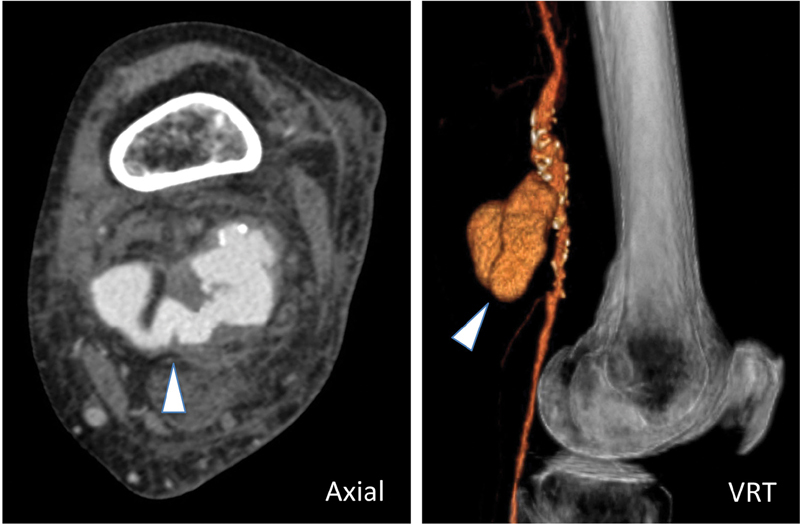
A voluminous popliteal artery aneurysm (arrowhead) with partial aneurysm sac thrombosis and signs of contained rupture. VRT, volume rendering technique.

Intraprocedural diagnostic digital subtraction angiography confirmed left popliteal artery aneurysm ( Fig. 2 ; arrowhead). A covered stent was deployed into the left popliteal artery segment with consequent aneurysm sac exclusion ( Fig. 3 ). The patient's symptoms resolved after the procedure with an uneventful postoperative course.

**Fig. 2:**
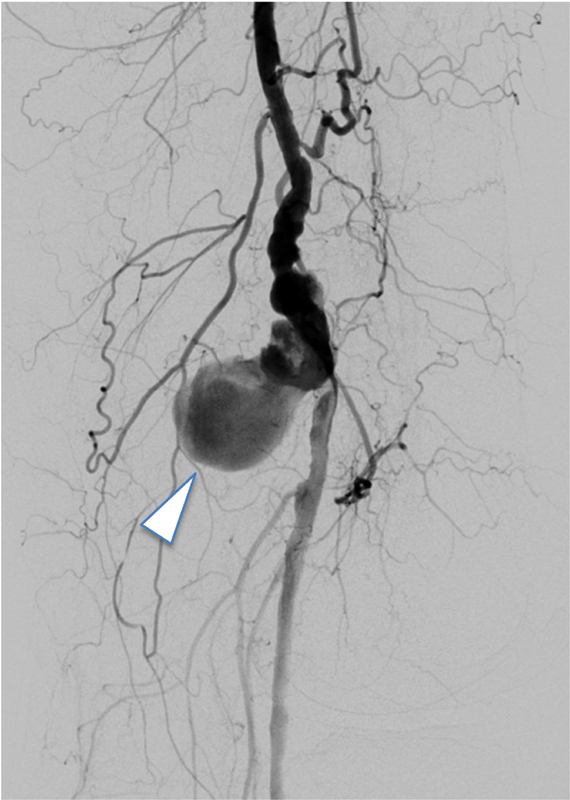
Intraprocedural diagnostic digital subtraction angiography confirmed left popliteal artery aneurysm (arrowhead).

**Fig. 3:**
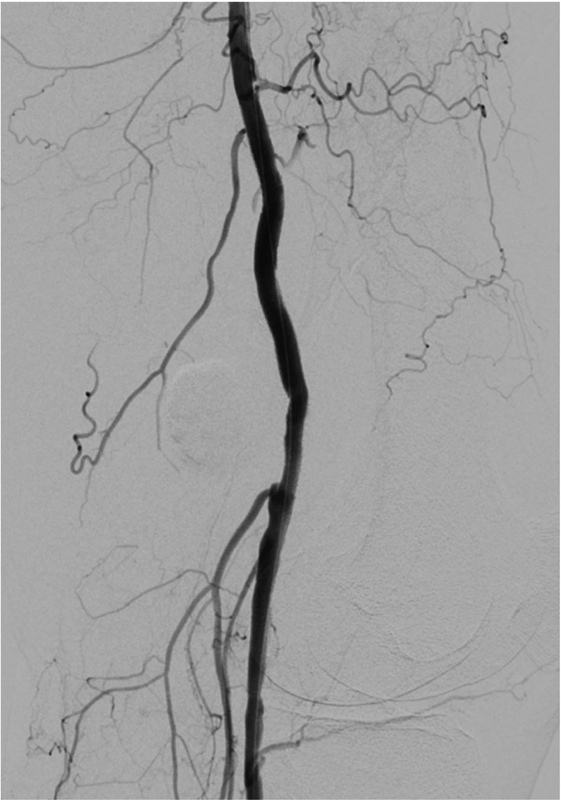
A covered stent was deployed into the left popliteal artery segment with consequent aneurysm sac exclusion.

Predischarge and follow-up ultrasound color Doppler confirmed thrombosis of the treated left popliteal aneurysm sac and stent lumen patency.

Ruptured popliteal artery aneurysm is an uncommon event in an uncommon disease. 1 Multidetector computed tomography in urgent cases is the diagnostic imaging of choice to evaluate aneurysm anatomy and possible complications, as well as for planning treatment approach. 1 2 In cases of ruptured popliteal artery aneurysm, as in our patient, with partial thrombosis and good run-off blood flow to the foot, an endovascular approach is indicated. 2 Endovascular deployment of a covered stent to exclude the popliteal artery aneurysm is a less invasive procedure compared with a conventional surgical approach. 3
